# Platelet-hemoglobin ratio predicts amputation in patients with below-knee peripheral arterial disease

**DOI:** 10.1186/s12872-022-02788-2

**Published:** 2022-07-28

**Authors:** Nail Burak Ozbeyaz, Gokhan Gokalp, Engin Algul, Haluk Furkan Sahan, Faruk Aydinyilmaz, Ilkin Guliyev, Kamuran Kalkan, Hilal Erken Pamukcu

**Affiliations:** 1 Department of Cardiology Clinic, Pursaklar State Hospital, 39 Cagatay Street, Pursaklar, Mimar Sinan District, 06145 Ankara, Turkey; 2 Diskapi Yildirim Beyazit Training and Research Hospital, University of Health Sciences, Ankara, Turkey; 3 Erzurum Education and Research Hospital, Erzurum, Turkey; 4 Medical Park Hospital, Tokat, Turkey

**Keywords:** Peripheral arterial disease, Below-knee, Amputation, Platelet-hemoglobin ratio

## Abstract

**Background:**

Peripheral arterial disease (PAD) causes significant morbidity today. Atherosclerosis is evident in the pathophysiological process in most patients, so PAD has similar risk factors as coronary artery disease. Platelet-Hemoglobin ratio (PHR) has been proven to predict mortality in atherosclerotic heart disease. We aimed to determine the relationship between PHR and below-knee amputation.

**Methods:**

The study is a single-center retrospective study. Platelet count/hemoglobin amount formula was used for PHR. Only PAD patients with below-knee critical extremity ischemia and unsuitable for revascularization were included in the study.

**Results:**

235 patients were included in the study retrospectively. The mean age was 65.7 ± 9.9 years and 175(74.5%) of them were male. In the amputated group, white blood cell, neutrophil, platelet, creatinine, glucose, and PHR were higher (p = .031, p = .045, p = .011, p = .048 p = .018, p = .004, respectively). Only hemoglobin values were lower (p = .003). Multivariable regression analysis showed; age, albumin and PHR were determined as independent risk factors for amputation (Age; OR (95%CI): (1.094(1.040–1.152), p = .001) (Albumin; OR (95% CI): 1.950(1.623–1.799), p = .001) (PHR; OR (95% CI): 1.872(1.246–2.812), p = .003). Receiver operating characteristics analysis performed to determine the optimal cut-off value of PHR for amputation, the optimal value was found 2.08 (65.8% sensitivity, 67.5% specificity, p < .001).

**Conclusions:**

PHR was a good predictor for BKA. Using the PHR, it may be possible to identify high-risk patients for amputation.

## Introduction

Below knee-peripheral arterial disease (BK-PAD) is seen at 12 to 20% in elderly individuals [1]. Especially hyperlipidemia (HL), hypertension (HT), diabetes mellitus (DM), chronic kidney disease (CKD), and smoking are the main risk factors in peripheral arterial disease (PAD) development. The presence of three or more of these risk factors increases the risk of PAD development by ten times [2]. All these risk factors are also found in coronary artery disease (CAD), that develops with almost the exact physio-pathological mechanisms, and PAD and CAD are often seen together [3].

Many parameters have been determined and evaluated on complete blood count to predict the development of adverse cardiac events in patients with CAD [4, 5]. Increased platelet count is associated with severe and poor cardiac outcomes in heart failure (HF) and CAD [6–8]. In addition, the presence of increased inflammatory activity and platelet activation in these patients were the main reasons for the poor results [9, 10]. However, low hemoglobin levels also cause poor cardiovascular effects [11, 12]. Here too, increased myocardial ischemia, neurohumoral activation, cardiac output, and abnormal myocardial remodeling seem to be the cause [13, 14]. Using the Platelet-Hemoglobin Ratio (PHR) parameter, which uses both platelet and hemoglobin values, will give more accurate and reliable results from them alone. For this purpose, no studies are showing whether PHR, which has proven predictive value in the follow-up of atherosclerotic cardiovascular diseases with recent studies [15, 16], will be helpful in the follow-up of below-knee (BK) PAD. In this context, we aimed to evaluate whether PHR can predict the possibility of below-knee amputation (BKA) in patients with BK-PAD who are not suitable for revascularization.

## Material-method

### Patient population

This study was conducted on patients who underwent peripheral angiography due to BK-PAD with critical limb threatening ischemia (CLTI) between July 2015 and August 2021 and who were not found suitable for endovascular and surgical revascularization (As the current guidelines indicate, it was decided that these patients cannot be revascularized with the decision of the joint council of the participants consisting of cardiology and cardiovascular surgery departments.). All patients had pain at rest (Rutherford class 3–4). The data of 1102 patients who underwent peripheral angiography were analyzed, 235 patients who met the criteria were included in the study. The patients were divided into two groups as amputated and non-amputee in their one-year follow-up after peripheral angiography. The exclusion criteria were an acute arterial disease, major tissue lost, active infection, hematological malignancy, cannot be given antiplatelet and statin therapy for any reason, having atrial fibrillation, use the anticoagulant treatment for any reason, having a cerebrovascular event, having severe valvular dysfunction, and patients with decompensated heart failure. In addition, patients who did not attend our clinic for follow-up were excluded from the study. BKA was defined as all amputations from any location below the knee. In the follow-ups, those who progressed to Rutherford class 5 and class 6 were applied BKA as a result of the consensus of the cardiology, cardiovascular surgery and orthopedic teams. It was conducted as a single-center, retrospective study. The data were accessed by scanning the hospital automation system and the patient files-records. The study protocol was made following the Declaration of Helsinki, with the approval of the local ethics committee.

### Laboratory and imaging data

The patient’s blood sample was taken from the peripheral venous route 24 h before the procedure. In laboratory examinations, whole blood tests, fasting glucose levels, lipid profile, liver, kidney, and thyroid functions were examined. The whole blood test was analyzed using an automatic complete blood analyzer (Symex XN-550 analyzer, Symex, Kobe, Japan) and other biochemical tests using standard biochemical techniques with Beckman Coulter LH 780 device (Beckman Coulter Inc., Brea, New York, USA).

### Definitions

PHR was produced by calculating the platelet count/hemoglobin count, neutrophil–lymphocyte ratio (NLR) = neutrophil count/platelet count, platelet-lymphocyte ratio (PLR) = platelet/lymphocyte count formulas [16]. Patients were classified according to the definition of the American Diabetes Society as the criteria for DM and based on the data of the ESC hypertension guideline as to the meaning of HT [17].

### Statistics

Categorical data are presented as numbers and percentages. For non-parametric data analysis, the chi-square test was used. All the variables were examined with the Kolmogorov–Smirnov test for normality and the Levene test for homogeneity of variances before significance tests were used. Normally distributed homogeneous data were used with the t-test in independent groups. The Mann–Whitney U test was used to evaluate the difference in the parameters that did not show normal distribution. Univariate and multivariate logistic regression was performed to analyze the defined risk factors for amputation development and determine independent risk factors. Receiver operating characteristic (ROC) analysis was used to estimate the optimal cut-off value of PHR in indicating BKA. Sensitivity, specificity, and area under the curve (AUC) values were calculated. All analyzes were performed with IBM SPSS 23.0 statistical package program (IBM Corp., Armonk, NY, USA). The significance level was considered p < 0.05 2- sided for all statistical analyses.

## Results

A total of 235 patients with BK CLTI that could not be revascularized were included in the study. The patients were divided into amputated (n = 37) and non-amputee (n = 198) groups within one year. The median amputations of the patients after angiography were determined as 209.5 (IQR (125–295)) days. The mean age of the patients was 65.7 ± 9.9 years, and the mean age of the patients in the amputated group was more advanced (71.0 ± 10.3 vs. 64.7 ± 9.5, p = 0.001). Other demographic findings were similar (Table 1).

**Table 1:** Basal demographic and laboratory characteristics of the patients according to amputation status

*Demographics*	Overall n = 235	Amputees n = 37	Non-amputee n = 198	p-value
Age (years), Mean(SD)	65.7 ± 9.9	71.0 ± 10.3	64.7 ± 9.5	**.001**
Male gender n (%)	175(74.5)	25(67.6)	150(76.1)	.18
Smoking n (%)	126(53.7)	22(57.9)	104(53.1)	.35
Dyslipidemia n (%)	118(50.2)	14(36.8)	104(52.8)	.079
DM n (%)	147(62.6)	19(50.3)	128(65.1)	.09
HT n (%)	133(56.6)	26(68.14)	107(54.3)	.076
CAD n (%)	69(29.4)	10(26.3)	59(30.1)	.702
*Laboratory parameters*				
Hemoglobin (g/l)	129.1 ± 25.7	118.0 ± 23.2	131.3 ± 25.6	**.003**
WBC (103/μl)	9.3 ± 3.1	9.4 ± 3.2	8.5 ± 2.0	**.031**
Neutrophil (10^3^/μl)	6.2 ± 2.8	6.4 ± 3.0	5.6 ± 1.8	**.045**
Lymphocyte (10^3^/μl)	1.9 ± 0.7	1.9 ± 0.6	1.9 ± 0.7	.67
Platelets, mm3	269.5 ± 102.8	314.3 ± 116.0	260.0 ± 98.0	**.011**
RDW	15.2 ± 2.4	15.2 ± 1.8	15.2 ± 2.5	.89
Total cholesterol (mg/dl)	177.5 ± 51.9	177.9 ± 53.0	175.3 ± 46.2	.77
LDL-C (mg/dl)	123.3 ± 40.7	125.5 ± 38.1	122.9 ± 41.2	.71
HDL-C (mg/dl)	39.1 ± 13.3	39.4 ± 10.7	39.0 ± 13.8	.86
Triglycerides (mg/dl)	165.2 ± 94.8	154.4 ± 98.4	167.3 ± 94.1	.46
Creatinine (mg/dl)	1.4 ± 1.3	1.4 ± 1.3	1.1 ± 0.8	**.048**
BUN (mg/dl)	50.5 ± 42.2	50.8 ± 44.8	48.8 ± 25.4	.70
Glucose (mg/dl)	151.1 ± 83.0	155.3 ± 87.3	129.3 ± 51.9	**.018**
Albumin g/dL	22.9 ± 17.3	15.0 ± 16.0	24.5 ± 17.2	**.004**
PHR	2.19 ± 1.10	2.82 ± 1.45	2.07 ± 0.97	**.004**

The values of which *p* values are indicated in bold are found statistically significant

*DM* Diabetes mellitus, *HT* Hypertension, *CAD* Coronary artery disease, *WBC* White blood cell, *RDW* red cell distribution width *LDL* Low-density lipoprotein, *HDL* high-density Lipoprotein, *PHR* Platelet Hemoglobin Ratio

**Fig. 1:**
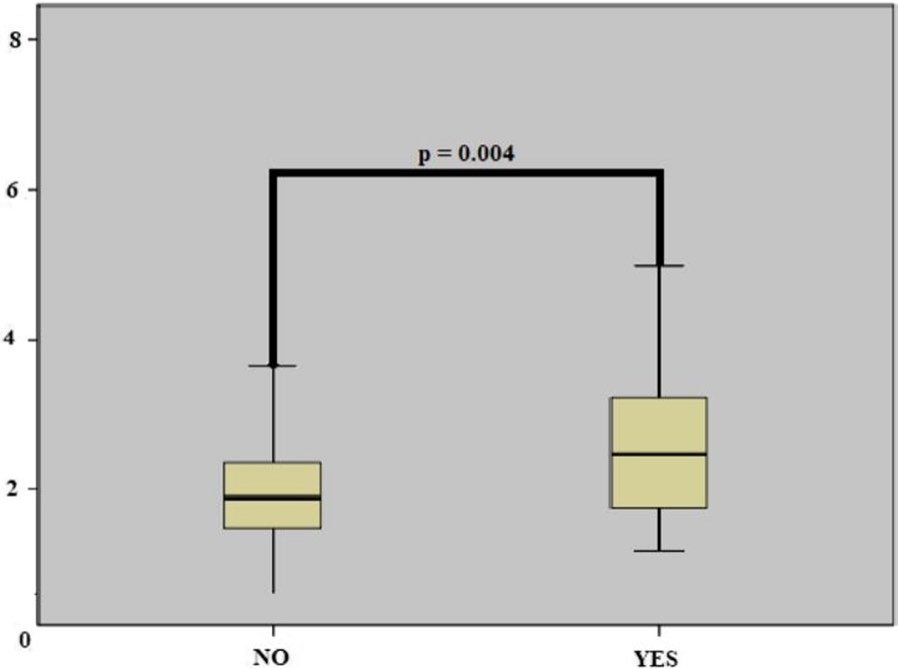
Displaying platelet-hemoglobin ratio (PHR) with the box-plot table when they are divided into two groups as amputee and non-amputee

When the laboratory parameters were compared, white blood cell (WBC), neutrophil, platelet, creatinine, and glucose were higher in the amputated group. Again, the PHR value was higher in the amputated group (2.82 ± 1.45 vs. 2.07 ± 0.97, p = 0.004) (Fig. 1). Conversely, in the amputee group, albumin (15.0 ± 16.0 g/dL vs. 24.5 ± 17.2 g/dL, p = 0.004) and hemoglobin (118.0 ± 23.2 g/l vs. 131.3 ± 25.6 g/l, p = 0.003) values were found to be lower than the non-amputated group. There was no difference between the groups in other laboratory parameters (Table1).

**Fig. 2:**
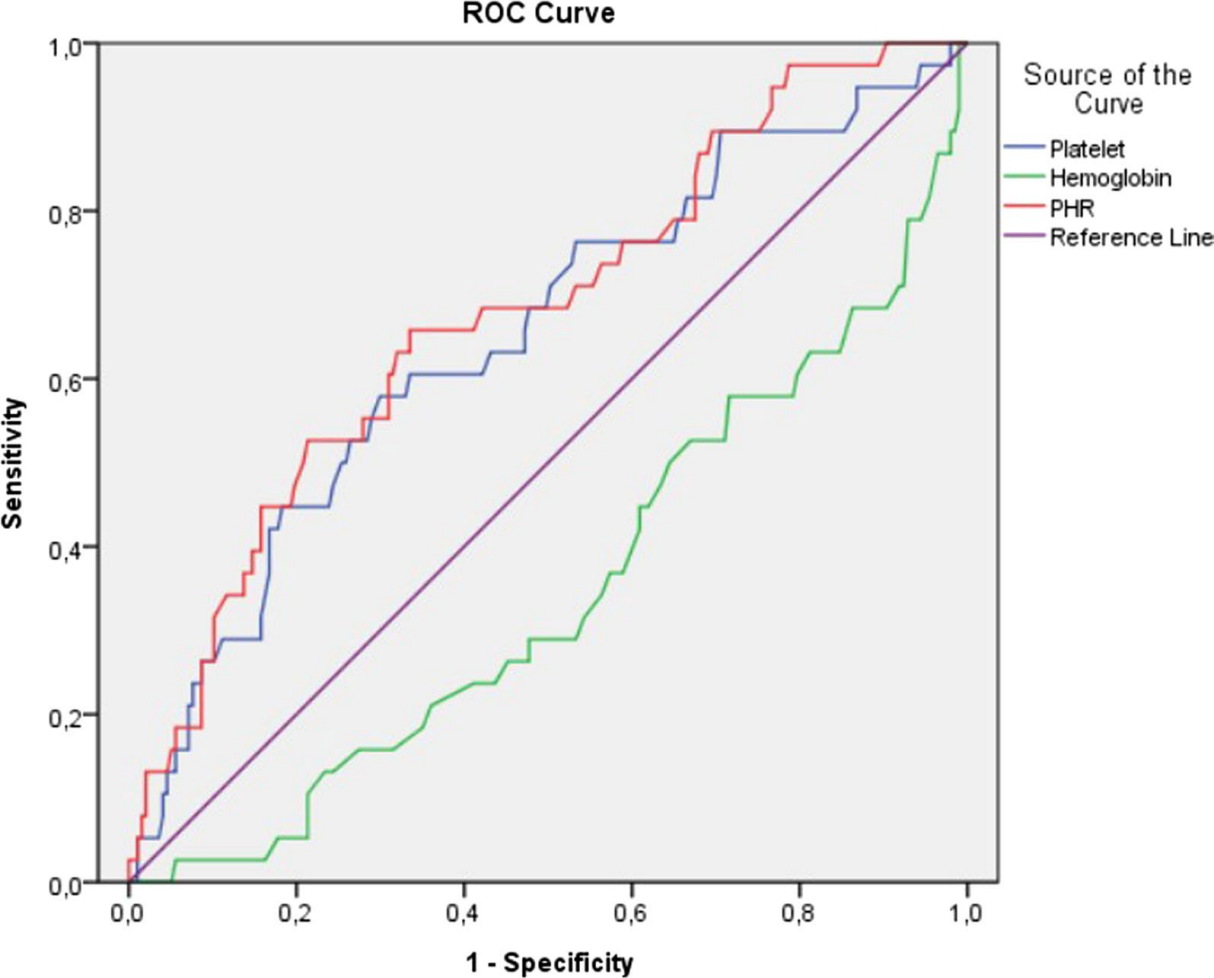
Receiver operating characteristics (ROC) curve for platelet-hemoglobin ratio (PHR), platelets and hemoglobin in below-knee amputation patients

ROC analysis was performed to determine the optimal PHR value for predicting the development of BKA within one year. A value of 2.08 was found as the optimal cut-off value (AUC:0.680(586–774), 65.8% Sensitivity, 67.5% Specificity, p < 0.001) (Table 2) (Fig. 2).

**Table 2:** Receiver operating characteristic curve analysis results to determine the value of PHR, platelets, and hemoglobins in predicting amputation

Risk factor	AUC (95%)	Cut-off	p	Sensitivity (%)	Specificity (%)
PHR	0.680(0.586–0.774)	2.08	< .001	65.8	67.5
Platelet	0.651(0.552–0.750)	257	.003	63.2	62.8
Hemoglobin	0.650(0.550–0.747)	132.5	.003	58.9	61.6

*PHR* Platelet-hemoglobin ratio, *AUC* Area under the curve

The univariate regression analysis results showed that age, hemoglobin, platelet, albumin, and PHR were good predictors for BKA. The statistically significant parameters between the groups were evaluated as predictor factors. In multivariate regression analyses; age, albumin and PHR were found to be independent risk factors for BKA (Age; OR (95%CI): (1.094(1.040–1.152), p = 0.001) (Albumin; OR (95% CI): 1.950(1.623–1.979), p = 0.001) (PHR; OR (95% CI): 1.872(1.246–2.812), p = 0.003) (Table 3).

**Table 3:** Regression analysis results in predictive factors for amputation

	Univariable analysis		Multivariable analysis	
	OR (95% CI)	*p*	OR (95% CI)	*p*
Age	1.073(1.032–1.116)	** < .001**	1.094(1.040–1.152)	**.001**
Hemoglobin	1.976(1.962–1.991)	**.002**	1.962(1.916–2.009)	.205
Platelet	2.003(2.001–2.007)	**.005**	2.014(1.993–2.035)	.114
Neutrophil	1.026(1.012–1.053)	**.003**	0.883(0.749–1.042)	.141
WBC	0.883(0.761–1.025)	.44	0.923(0.776–1.097)	.36
Creatinine	0.717(0.442–1.164)	.17	0.725(0.417–1.260)	.25
Glucose	0.995(0.990–1.001)	.09	0.995(0.988–1.002)	.99
Albumin	1.368(1.245–1.690)	**.005**	1.250(1.123–1.579)	**.001**
PHR	1.439(1.042–1.963)	** < .001**	1.472(1.106–1.912)	**.003**

The values of which *p* values are indicated in bold are found statistically significant

*WBC* White Blood Cell, *PHR* Platelet hemoglobin Ratio

## Discussion

To our knowledge, this is the first study to investigate the predictive value of the PHR parameter in BK-PAD patients for BKA, which was previously proven to be predictive of coronary artery diseases and heart failure [15, 16]. Our study showed that the PHR value was significantly higher in amputated patients (2.82 ± 1.45 vs. 2.07 ± 0.97, p = 0.004) and was a strong independent risk factor for the development of BKA (1.872, OR(95)%) (1.246–2.812), p = 0.003).

Platelet count elevation and anemia are clinical factors that cause a poor prognosis for peripheral arterial disease [18–20]. The presence of anemia adversely affects the prognosis of PAD in several ways. Of these, direct extremity ischemia due to anemia is the leading cause. In addition, anemia may increase the preload and decrease the afterload in the chronic period, resulting in relative heart failure and overt heart failure. The end-organ perfusion defect, which develops due to heart failure, also increases extremity ischemia [18, 21, 22]. At the same time, the atherosclerotic process in the extremity is accelerated due to increased systemic inflammation due to anemia. This condition exacerbates lower extremity ischemia and contributes to poor outcomes [23]. As shown in many previous studies, platelet count elevation increases inflammation, accelerates and advances the atherosclerotic process, and induces thrombotic processes, which causes many cardiovascular adverse outcomes [7–10, 24]. All this information shows that anemia and high platelet counts are poor prognostic indicators for vascular diseases. The combination of both may be much more valuable for this patient group than the use of platelet alone or hemoglobin alone. When all these data are evaluated, the high PHR value can be considered both a cause and a result of this clinical picture. This situation supports the argument that PHR is an important prognostic indicator for atherosclerotic arterial disease developing in the lower extremities in many respects. Similarly, according to the statistical analysis, the high PHR value in our study is much more meaningful in showing the development of amputation than the platelet and hemoglobin values alone. The fact that PHR is an independent risk factor with age due to the regression analysis clearly shows this relation.

In our study, amputation development was observed more frequently in patients with high leukocyte and neutrophil counts. These data are consistent with the information presented by previous studies in the literature showing that increased inflammation worsens the atherosclerotic process [24, 25]. Here, the fundamental pathology should be evaluated bilaterally; firstly, there is an increase in the inflammatory response resulting from ischemia in the lower extremities, which further accelerates atherosclerosis and increases the already existing critical extremity ischemia leading to a vicious circle. Secondly, due to ischemia and the prolongation of the recovery period, lower extremity infections (LEI) occur. LEI increases the existing inflammation, delays the disorder's healing, and even leads to amputation without recovery [2, 19, 24, 26].

CKD is one of the long-known, well-defined significant risk factors for cardiovascular diseases due to the faster progression of atherosclerosis [27]. Increased accumulation of uremic toxins, oxidative stress, and inflammation, overactivation of the renin–angiotensin–aldosterone system systemically exacerbate atherosclerosis in this patient group. As a result, PAD development and complexity progress fastly. In this patient group, stenosis, thrombotic events, critical extremity ischemia, and eventually amputation is related to mortality [28]. In our study, the creatinine level was significantly higher in amputee patients. This result supports previous experiences and studies. Since CKD patients have an increased risk of developing cardiovascular diseases, it is essential not to overlook the screening activities to be carried out in this patient group. Early and aggressive treatments with the recognition of atherosclerotic processes when not yet critical may prevent amputation in these patients early.

In our study, although diabetic individuals did not show a statistically significant difference between amputees and non-amputees, amputation was significantly more common in the group with high fasting glucose. Tong Zhao et al. evaluated the complexity of the coronary arteries of patients without DM in a previous study. They found that patients with high fasting glucose had more complex coronary artery diseases even if they did not have diabetes. This result shows that hyperglycemia is toxic to arterial structures independent of insulin and increases atherosclerosis. Here, atherosclerosis increases and accelerates with hyperglycemia; increased inflammation, oxidative stress, and different metabolites caused by all these seem to be responsible [29]. As a result, it is understood that strict blood sugar control in patients with PAD, even in the absence of DM, can reduce vascular morbidity and mortality that will develop in the future.

Albumin; is an essential protein that provides oncotic pressure in the body, transports many agents in the blood (fatty acids, bile acids, etc.), neutralizes free oxygen radicals by keeping them, and has antiplatelet activity [30]. Along with all these, albumin also shows the nutritional status. Many previous studies have shown that surgical mortality and mortality due to other diseases are much higher in patients with low albumin levels [31]. Congcong Ding et al. showed that peripheral artery disease is more common in male patients with low albumin levels [32]. Again, recent studies have shown that low prognostic nutritional index (PNI), a marker-based on albumin and neutrophils, in patients with limb ischemia causes more critical limb ischemia and amputations [33, 34]. Similar to mentioned studies, our study showed that albumin is one of the risk factors considered in individuals with atherosclerotic lower extremity disease. In particular, the lack of albumin indicates nutritional deficiency. It is possible to prevent the progression of the diseases and the worse outcome that will arise with careful dietary supplements for this patient group. At the same time, the increase in systemic inflammation of patients with low albumin levels and malnutrition promotes atherosclerosis, also known [35, 36].

## Limitations

The study is a single-center, retrospective study. The number of patients is relatively small. The presence of CLTI in patients was evaluated only according to symptoms and conventional peripheral angiography results. Objective measurement methods such as ABPI were not used. Traditional inflammatory markers such as CRP have not been evaluated in patients. These results can be reconsidered with future studies in which modern revascularization practices and supportive treatments are evaluated together.

## Conclusion

PHR was a good predictor for BKA. It is easy to reach the PHR parameter with whole blood analysis, routinely evaluated, and easy to access, especially clinical applications. PHR calculation can contribute to determining the risk status of BK-PAD quickly and easily. A larger patient population and prospective studies are needed to confirm the results of this study.

## Data Availability

The datasets used and/or analyzed during the current study are available from the corresponding author on reasonable request.

## Abbreviations

BK-PAD: Below knee-peripheral arterial disease
HL: Hyperlipidemia
HT: Hypertension
DM: Diabetes mellitus
CKD: Chronic kidney disease
CAD: Coronary artery disease
HF: Heart failure
PHR: Platelet-hemoglobin ratio
BKA: Below-knee amputation
CLTI: Critical limb threating ischemia
NLR: Neutrophil–lymphocyte ratio
PLR: Platelet-lymphocyte ratio
ROC: Receiver operating characteristic
AUC: Area under the curve
LEI: Lower extremity infections
